# The Effects of New Selective PPAR*α* Agonist CP775146 on Systematic Lipid Metabolism in Obese Mice and Its Potential Mechanism

**DOI:** 10.1155/2020/4179852

**Published:** 2020-05-04

**Authors:** Shengjie Tang, Fang Wu, Xihua Lin, Weiwei Gui, Fenping Zheng, Hong Li

**Affiliations:** ^1^Department of Endocrinology, The Affiliated Sir Run Run Shaw Hospital, School of Medicine, Zhejiang University, Hangzhou, China 310016; ^2^Biomedical Research Center and Key Laboratory of Biotherapy of Zhejiang Province, The Affiliated Sir Run Run Shaw Hospital, Zhejiang University, Hangzhou, Zhejiang, China 310016

## Abstract

**Purpose:**

Peroxisome proliferator-activated receptor *α* (PPAR*α*) plays a crucial role in the control of lipid homeostasis. Here, we investigated the effects of CP775146, a new selective PPAR*α* agonist, on lipid metabolism in diet-induced obese mice and its possible mechanism.

**Methods:**

C57BL/6 mice were fed a high-fat diet (HFD) for 12 weeks to induce obesity and then received CP775146 via intraperitoneal injection for 3 days. The content/morphology of the liver, serum lipid, and liver function was measured. The expression of genes related to lipolysis and synthesis in liver was detected by quantitative real-time PCR (qRT-PCR).

**Results:**

The safe dose of CP775146 was <0.3 mg/kg. CP775146 reduced the serum levels of liver enzymes, such as alanine aminotransferase (ALT) and glutamic-oxaloacetic transaminase (AST) and lipid metabolism-related biomarkers, including triglycerides (TGs) and low-density lipoprotein cholesterol (LDL-c), non-high-density lipoprotein cholesterol (non-HDL-c), and hepatic TG content, at a dosage of 0.1 mg/kg. HFD-induced pathological liver changes improved after CP775146 treatment. The expression of genes involved in liver fatty acid oxidation (acyl-coenzyme A dehydrogenase, long chain (*Acadl*), acyl-CoA oxidase 1 (*Acox*-*1*), carnitine palmitoyltransferase-1 (*CPT*-*1*), and enoyl-CoA, hydratase/3-hydroxyacyl CoA dehydrogenase (*Ehhadh*)) was upregulated in CP775146-treated mice. Furthermore, CP775146 induced the expression of thermogenesis genes (cell death-inducing DFFA-like effector a (*Cidea*), uncoupling protein 1 (*Ucp1*)) and lipolysis genes (hormone-sensitive lipase (*Hsl*), adipose tissue triglyceride lipase (*Atgl*)) in epididymal white adipose tissue (eWAT), activating browning and thermogenesis.

**Conclusion:**

CP775146 efficiently alleviates obesity-induced liver damage, prevents lipid accumulation by activating the liver fatty acid *β*-oxidation pathway, and regulates the expression of genes that control brown fat-like pathway in eWAT.

## 1. Introduction

Dyslipidemic conditions are characterized by increased levels of blood free fatty acid (FFA), triglyceride (TG), total cholesterol (TC), and low-density lipoprotein cholesterol (LDL-c) and decreased levels of high-density lipoprotein cholesterol (HDL-c) [1]. Dyslipidemia has been linked to the increased prevalence of diabetes, cardiovascular diseases, and fatty liver diseases [2–4]. In addition to genetic factors associated with familial lipid disorders, dyslipidemia is closely linked to the dysregulation of nutrient homeostasis, which defines obesity. Abnormal adipose tissues secrete several adipokines and produce excessive FFAs, thereby enhancing dyslipidemia [5]. Hence, lipid control therapies are urgently required.

Peroxisome proliferator-activated receptors (PPARs) are members of the superfamily of nuclear hormone receptors that were first discovered in Xenopus [6]. Upon ligand binding, PPARs (PPAR*α*, PPAR*γ*, and PPAR*δ*/*β*) form heterodimers with retinoid X receptors and then interact with PPAR response elements to regulate target gene expression [7]. The three PPAR subtypes are expressed differentially in various tissues, thereby allowing selective changes in the expression levels of genes related to lipid and glucose metabolism. Tissues actively engage in fatty acid metabolism, such as the liver, brown adipose tissue (BAT), and the heart [8], and express high levels of PPAR*α*, which play major roles in fatty acid uptake and activation of mitochondrial *β*-oxidation, glucose metabolism, and hepatic acute phase response [9]. PPAR*α* regulates the expression of genes encoding the rate-limiting enzymes of peroxisomal *β*-oxidation, including *Acox1* and *Ehhadh*. Fatty acid transport across the mitochondrial membrane is triggered by PPAR*α*-dependent regulation of carnitine palmitoyltransferase-1 (*CPT*-*1*) [10]. Thus, PPAR*α* is the primary target of hypolipidemic drugs, including fibrates (e.g., gemfibrozil, bezafibrate, and fenofibrate). However, these agents are weak and relatively poorly selective PPAR agonists and often trigger muscle or cardiac toxicities [11]. An efficient and selective PPAR*α* agonist is urgently required.

CP775146 is a novel piperidine-based PPAR*α* agonist that binds more strongly and selectively to the PPAR*α* ligand than classical fibrates. CP775146 exhibited outstanding TG-lowering activity in chow-fed mice and activated transcriptional networks triggering PPAR*α*-mediated induction of fatty acid oxidation and anti-inflammatory activities [12]. However, this drug is used to treat patients with hyperlipidemia. The effects of CP775146 on lipid metabolism in obese mice remain unknown.

This study was conducted to evaluate the effects of CP775146 on systematic lipid metabolism and its potential mechanism, in diet-induced obese mice and focused on PPAR*α* target tissues.

## 2. Materials and Methods

### 2.1. Animals, Diets, and Treatments

Four-week-old male C57BL/6 mice were purchased from the Slack Experimental Animal Center of the Chinese Academy of Sciences (Shanghai, China). Mice were housed at 22°C under a 12 h/12 h light/dark cycle and given free access to water and standard chow (63.92% carbohydrates, 26.18% protein, and 9.9% fat) or a high-fat diet (HFD; 35% carbohydrates, 20% protein, and 45% fat) for 12 weeks. The safe dose of CP775146 was determined. Chow-fed mice were randomized into the following groups: (1) chow-control (*n* = 4) and (2) chow-CP775146 groups (0.3, 0.6, 1.0, 1.2, or 1.5 mg/kg); *n* = 4/group. According to the safe dose, HFD-fed mice were also divided into the following groups: (1) HFD-NC (*n* = 4), (2) HFD-0.1 (0.1 mg/kg, n = 4), and (3) HFD-0.3 (0.3 mg/kg, *n* = 4). Saline or CP775146 was given via intraperitoneal injection for 3 days. After fasting for 12 h since the last drug treatment, all mice were killed via CO_2_ asphyxiation. Trunk blood was collected for biochemical analyses. The selective PPAR*α* agonist CP775146 (purity ≥ 98%) was obtained from Sigma Chemical Co. (St. Louis, MO, USA).

All animal experimental procedures were approved by the Animal Welfare Ethics Committee of Zhejiang University.

### 2.2. Serum Biochemical Measurements

Blood was held at room temperature for 30 min and centrifuged to obtain serum, which was stored at –80°C prior to analysis. The levels of TC, TG, LDL-c, HDL-c, and liver enzyme were measured automatically (Hitachi 7020, Japan). Non-HDL-c values were calculated as follows: non − HDL − c = TC–(HDL − c).

### 2.3. Histological Study of Liver Tissue and Epididymal White Adipose Tissue (eWAT)

Liver tissue and eWAT were weighed and fixed. The samples were then embedded in paraffin blocks, sectioned at a thickness of 5 *μ*m, stained with hematoxylin and eosin (H&E), and observed under a light microscope.

### 2.4. Hepatic TG and TC Measurements

Hepatic TG and TC levels were measured using 100 mg of frozen liver samples. The tissues were homogenized in 1 mL of phosphate-buffered saline (1x) and centrifuged at 2,500 rpm. The supernatant (20 *μ*L) was assayed using commercial kits (Nanjing Jiancheng, China) according to the manufacturer's protocols.

### 2.5. Quantitative Real-Time PCR

Total RNA was extracted from liver tissues by using RNAiso Plus (Takara Bio Inc., Shiga, Japan) according to the manufacturer's instructions and stored at –80°C prior to analysis. First-strand cDNA synthesis was performed according to the manufacturer's instructions, followed by PCR. Data were quantitated using the relative mRNA expression ratio (2^−*ΔΔ*ct^ method). Primer sequences are listed in Table 1.

### 2.6. Western Blot Analysis

Total protein was isolated from the liver in a lysis buffer for 30 min at 4°C. Protein concentration was measured using BCA Protein Assay Reagent (P0011, Beyotime Biotechnology, China). The proteins were transferred to a polyvinylidene difluoride membrane, blocked with 5% nonfat dry milk in PBS with 0.02% (*v*/*v*) Tween-20, and incubated with primary antibodies at 4°C overnight. The membrane was washed and incubated for 1 h at room temperature with a peroxidase-labeled secondary antibody. After washing, protein bands were visualized by electrochemiluminescence (FD8030, FDBio Science, China). Anti-Pgc1*β* (A17089), Cpt1*α* (A5307), and Cpt1*β* (A6796) were obtained from ABclonal (Wuhan, China). Mouse anti-*β*-actin antibody (A5441) was purchased from Sigma-Aldrich (St. Louis, MO, USA).

### 2.7. Statistical Analysis

Data are presented as means ± SD. Statistical analysis was conducted with SPSS version 20.0 software by using one-way ANOVA for multiple group comparisons or Student's *t*-test for two-group comparisons. *P* values < 0.05 indicated statistical significance.

## 3. Results

### 3.1. CP775146 Attenuates Dyslipidemia in HFD-Induced Obese Mice

**Figure 1:**
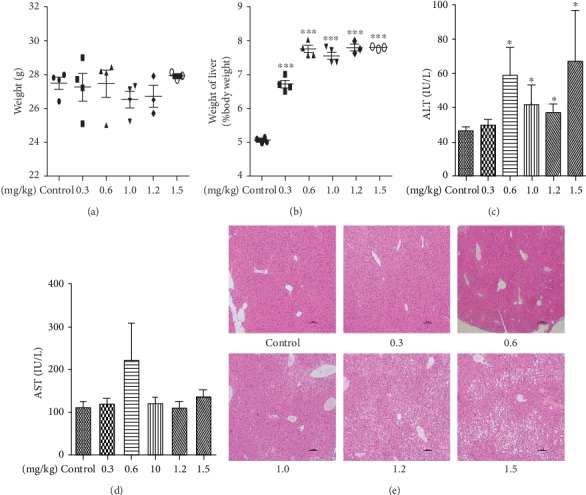
The safe dose of CP775146. (a) Body weight, (b) liver/weight ratio, (c) ALT level, and (d) AST level. (e) H&E staining of the liver sections (scale bar = 100 *μ*m). Means ± SDs (*n* = 4/group) are shown. ^∗^*P* < 0.05,^∗∗^*P* < 0.01, and ^∗∗∗^*P* < 0.001 vs. the chow-fed control group.

Chow-fed mice were treated with different doses of CP775146. CP775146 increased the liver weight but did not affect the on mean body weight (Figures 1(a) and 1(b)). At >0.3 mg/kg, CP775146 significantly increased the serum alanine aminotransferase (ALT) level (a measure of liver function, Figure 1(c)) but not that of aspartate aminotransferase (AST, Figure 1(d)). H&E staining showed that the hepatocytes of the groups (>0.3 mg/kg) contained more lipid droplets and exhibited more ballooning-induced degeneration than those of the control and 0.3 mg/kg group, respectively (Figure 1(e)). Thus, CP775146 at >0.3 mg/kg was hepatotoxic.

**Figure 2:**
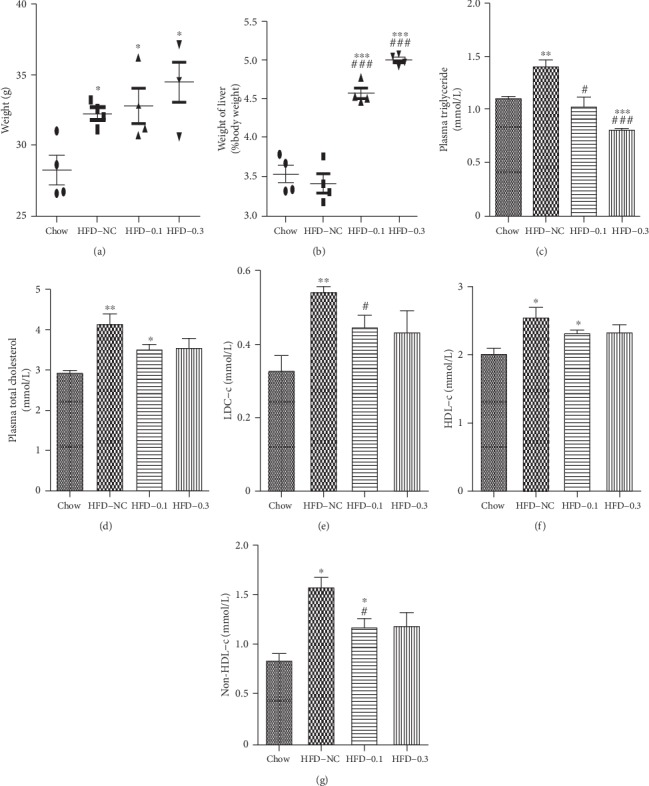
CP775146 attenuates dyslipidemia in HFD mice. (a) Body weight, (b) liver/weight ratio, (c) serum TG level, (d) serum TC level, (e) serum LDL-c level, (f) serum HDL-c level, and (g) serum non-HDL-c level. Means ± SDs (*n* = 4/group) are shown. ^∗^*P* < 0.05, ^∗∗^*P* < 0.01, and ^∗∗∗^*P* < 0.001 vs. chow-fed mice; ^#^*P* < 0.05, ^##^*P* < 0.01, and ^###^*P* < 0.001 vs. the HFD-NC group. According to the safe dose, HFD-fed mice were divided into these groups: (1) HFD-NC group, *n* = 4; (2) HFD-0.1 group (0.1 mg/kg), *n* = 4; and (3) HFD-0.3 group (0.3 mg/kg), *n* = 4. Saline or CP775146 was given via intraperitoneal injection for 3 days.

The mean body weight (Figure 2(a)) of the HFD mice was higher than that of chow-fed mice, but CP775146 had no effect on the body weight. CP775146-treated mice exhibited significantly higher liver/body weight ratio (Figure 2(b)) than the HFD-NC group. In terms of serum biochemical parameters (Figures 2(c)–2(f)), HFD-NC mice had higher TG, TC, LDL-c, HDL-c, and non-HDL-c levels than chow-fed mice did (Figures 2(c)–2(g)). The plasma levels of TG, LDL-c, and non-HDL-c in the HFD-CP775146 group (Figures 2(c), 2(e), and 2(g), respectively) were significantly lower than those tin the HFD-NC group in a CP775146 dose-dependent manner. CP775146-treated mice exhibited a trend toward a decrease in the serum TC level (Figure 2(d)) but not HDL-c level (Figure 2(f)).

### 3.2. CP775146 Alleviates Hepatic Damage Induced by HFD

**Figure 3:**
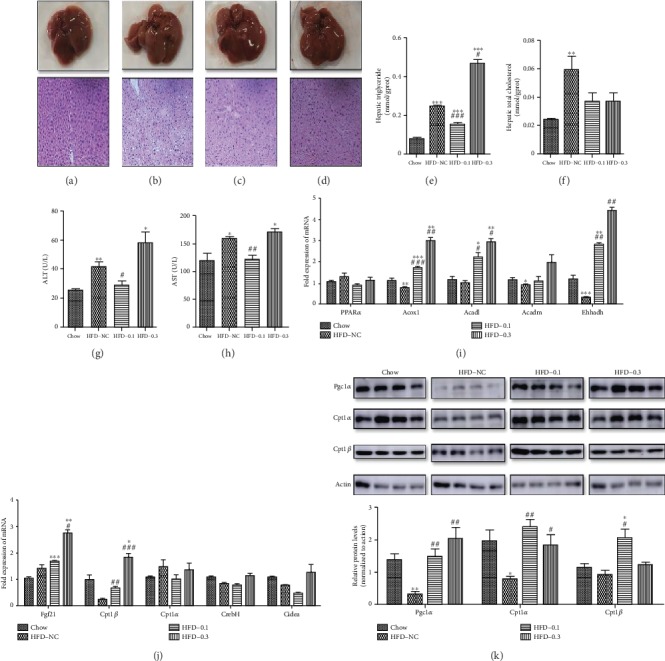
CP775146 alleviates hepatic damage induced by an HFD. H&E staining of liver sections (original magnification, 200x); *n* = 4/group. (a) Chow-fed group, (b) HFD-NC group, (c) HFD-CP775146 0.1 mg/kg group, and (d) HFD-CP775146 0.3 mg/kg group. (e) TG and (f) TC liver levels, (g) ALT levels, and (h) AST levels. (i, j) The mRNA expression levels of various genes. Means ± SDs are shown (*n* = 4/group). (K1, K2) The protein expression levels of various genes. ^∗^*P* < 0.05, ^∗∗^*P* < 0.01, and ^∗∗∗^*P* < 0.001 vs. the chow-fed group; ^#^*P* < 0.05, ^##^*P* < 0.01, and ^###^*P* < 0.001 vs. HFD-NC group.

H&E staining showed that hepatocytes in the HFD-NC group (Figure 3(b)) were larger than those in the chow-fed group (Figure 3(a)), contained increased amounts of lipid droplets, and exhibited additional ballooning-induced degeneration (Figure 3(b)). CP775146 alleviated HFD-induced pathological liver changes (Figures 3(c) and 3(d)). CP775146 reduced the hepatic TG levels at a lower dose (0.1 mg/kg) than those in HFD-NC mice (Figure 3(e)). CP775146 tended to decrease the hepatic TC levels (Figure 3(f)) but not significantly. HFD-NC mice exhibited increased serum ALT and AST levels relative to chow-fed mice (Figures 3(g) and 3(h)). CP775146 at 0.1 mg/kg significantly inhibited HFD-induced increases in ALT and AST levels (Figures 3(g)and 3(h)).

### 3.3. CP775146 Affects the Expression of Genes Involved in Liver Lipid Metabolism

CP775146 did not affect the liver level of mRNA encoding PPAR*α* compared with that in the HFD-NC group. However, CP775146 significantly increased the expression levels of PPAR*α* target genes responsible for fatty acid oxidation, including *Acadl*, *Acox1*, and *Ehhadh*(Figure 3(i)). CP775146 significantly increased the expression levels of *Fgf21* and *Cpt1β* (Figure 3(j)). CP775146 also upregulated the protein levels of Cpt1*α* and *Cpt1β* (Figures 3, K1 and K2). Thus, CP775146 activated fatty acid oxidation and PPAR*α* signaling in the livers of HFD-fed mice.

### 3.4. CP775146 Activates PPAR*α*-Mediated Gene Expression in eWAT of HFD-Fed Mice

**Figure 4:**
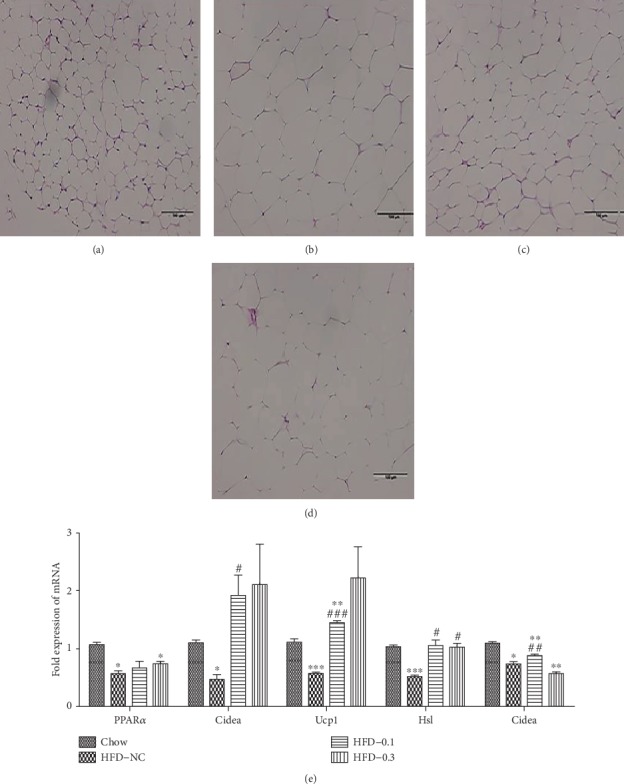
CP775146 activates PPAR*α*-mediated gene expression in the eWAT of mice fed an HFD. eWAT H&E staining data, scale bar = 100 *μ*m; *n* = 4/group. (a) Chow-fed group, (b) HFD-NC group, (c) HFD-CP775146 0.1 mg/kg group, and (d) HFD-CP775146 0.3 mg/kg group. (e) The expression levels of genes involved in thermogenesis and lipolysis. Means ± SDs are shown (*n* = 4/group). ^∗^*P* < 0.05, ^∗∗^*P* < 0.01, and ^∗∗∗^*P* < 0.001 vs. the chow-fed group; ^#^*P* < 0.05, ^##^*P* < 0.01, and ^###^*P* < 0.001 vs. HFD-NC group.

The H&E staining of the eWAT showed that 0.1 mg/kg CP775146 decreased the adipocyte sizes compared with HFD-NC mice (Figures 4(a)–4(c)), and no change was observed in the HFD-0.3 group (Figure 4(d)). In the eWAT, CP775146 significantly increased the expression levels of adipose TG lipase (*ATGL*) and hormone-sensitive lipase (*Hsl*; Figure 4(e)), thereby inducing lipolysis. CP775146 also upregulated the expression of genes involved in thermogenesis, including *Ucp1* and *Cidea* but did not change that of PPAR*α* compared with that in HFD-NC mice (Figure 4(e)).

## 4. Discussion

We explored the effects of CP775146, a new selective PPAR*α* agonist, on lipid metabolism in diet-induced obese mice. CP775146 significantly reduced the plasma TG and LDL-c levels and hepatic TG content at the dosage of 0.1 mg/kg. CP775146 reduced the plasma ALT levels and pathological liver changes, thereby alleviating hepatic damage induced by HFD. CP775146 activated the liver PPAR*α*-associated pathway of fatty acid oxidation and upregulated the expression genes involved in thermogenesis and lipolysis in eWAT. Hence, CP775146 improved systematic lipid metabolism in HFD-induced obese mice.

PPAR*α* is mainly expressed in the liver and plays a crucial role in hepatic physiology by regulating the balance between systematic fatty acid and TG metabolism [13]. Compared with classical PPAR*α* agonists, CP775146 is a selective PPAR*α* modulator that strongly and selectively stimulates PPAR*α*-related pathways [12]. CP775146 significantly upregulated the expression levels of the *Acox1*, *Acadl*, and *Ehhadh* genes involved in fatty acid oxidation and decreased plasma and hepatic TG levels. In the liver, adipose tissues, and normal lipogenic tissues, fatty acid pathways facilitate the storage of excess energy as TG, which is later used to supply energy. Fatty acid oxidation occurs in the mitochondria, and *CPT-1* is the key enzyme that regulates the entry of fatty acids into the mitochondria [14]. PPAR*α* upregulates *CPT-1* [15]. Mammals have three CPT1 isoforms (*CPT1A*, *CPT1B*, and *CPT1C*). *CPT1A* is enriched in the liver and plays a key role in fatty acid oxidation. *CPT1B* is mainly found in muscles, including cardiomyocytes, which are the major consumers of fatty acids. *CPT1C* is present in the brain and testis [16]. A previous study suggested that the three isoforms are confined to specific tissues [17]. CPT1A, as the liver isoform, catalyzes the rate-limiting step of converting acyl-coenzyme into acyl-carnitines, which can cross the membranes to enter the mitochondria in the fatty acid oxidation pathway [18]. CPT1A protects obese mice against hepatic steatosis and insulin resistance [19]. In the present study, the level of mRNA encoding *CPT1B* was downregulated in the liver of HFD-fed mice. CP775146 reversed this effect and upregulated the expression levels of genes that are involved in fatty acid oxidation in HFD-fed mice. The protein levels of Cpt1*α* and Cpt1*β* were also upregulated. *Cpt1β* may play a vital role in CP775146-mediated activation of fatty acid oxidation in the liver.

Adipocytes provide metabolic energy by balancing lipolysis and TG synthesis [20]. Lipolysis in the WAT is stimulated by *β*3 adrenergic receptor signaling, which activates lipolytic enzymes, including *HSL*, *ATGL*, and perilipin [21]. HSL plays a major role in the lipolysis of cellular fat stores [22]. The activation of thermogenic genes, including *Ucp1*, *Cidea*, and *Cpt1b*, converts white adipocytes into BAT-like beige adipocytes [23]. This activation process reduces WAT weight and lipid droplet size. In the present study, CP775146 activated the expression levels of eWAT genes that are involved in lipolysis (*Hsl* and *Atgl*) and thermogenesis (*Ucp1* and *Cidea*). The mechanism by which CP775146 decreases adipocyte sizes remains unclear.

CP775146 significantly increased the liver weight in chow- and HFD-fed mice. A previous study showed that the liver/body weight ratio increases significantly after gemfibrozil treatment. PPAR*α* activation plays a crucial role in hepatomegaly (pathological liver enlargement) induced by peroxisome proliferation [24, 25]. In the present study, the increase in the serum liver enzyme levels can be partly attributed to mitochondrial overload, which in turn reflected the overactivation of fatty acid oxidation. CP775146 efficacy was compromised by dose-related adverse effects, which have gained increasing research attention. Further studies are required to explore these adverse reactions and the mechanism underlying their prevention.

## 5. Conclusion

CP775146, a new selective PPAR*α* agonist, significantly reduces the levels of serum TG and liver enzymes, improves hepatic steatosis induced by HFD, and decreases adipocyte droplet sizes. The effects of CP775146 are partially mediated through its regulation of PPAR*α* target genes that are involved in fatty acid oxidation in the liver and lipolysis in eWAT. Further study on the mechanism of CP775146 on lipid metabolism may provide new sights into the clinical treatment of hyperlipidemia.

## Figures and Tables

**Table 1:** Primer sequences of real-time polymerase chain reaction.

Gene	Forward	Reverse
*Acadl*	AGGGTTTAGTTTTGAGTTGACGG	CCCCGCTTTTGTCATATTCCG
*Acadm*	TCTTTTCCTCGGAGCATGACA	GACCTCTCTACTCACTTCTCCAG
*Acox1*	CGATCCAGACTTCCAACATGAG	CCATGGTGGCACTCTTCTTAACA
*Cpt1α*	CTCCGCCTGAGCCATGAAG	CACCAGTGATGATGCCATTCT
*Cpt1b*	GCACACCAGGCAGTAGCTTT	CAGGAGTTGATTCCAGACAGGT
*Fgf21*	AGATCAGGGAGGATGGAACA	TCAAAGTGAGGCGATCCATA
*Ehhadh*	ATGGCTGAGTATC TGAGGCTG	ACCGTATGGTCCAAACTAGCTT
*CrebH*	CCTGTTTGATCGGCAGGAC	CGGGGGACGATAATGGAGA
*PPARα*	GCAGTGCCCTGAACATCGA	CGCCGAAAGAAGCCCTTAC
*Ucp1*	GGCATTCAGAGGCAAATCAGCT	CAATGAACACTGCCACACCTC
*Hsl*	GAGCGCTGGAGGAGTGTTTT	TGATGCAGAGATTCCCACCTG
*Atgl*	GGATGGCGGCATTTCA	CAAAGGGTTGGGTTGG
*Cidea*	GCAGCCTGCAGGAACTTATCAGC	GATCATGAAATGCGTGTTGTCC
*β-Actin*	GAGACCTTCAACACCCCAGC	ATGTCACGCACGATTTCCC

## Data Availability

The datasets used and/or analyzed during the present study are available from the corresponding author on reasonable request.

## Abbreviation

PPAR*α*: Peroxisome proliferator-activated receptor *α*
HFD: High-fat diet
ALT: Alanine aminotransferase
AST: Aspartate aminotransferase
TG: Triglyceride
TC: Total cholesterol
HDL-c: High-density lipoprotein cholesterol
LDL-c: Low-density lipoprotein cholesterol
Non-HDL-c: Non-high-density lipoprotein cholesterol
Acox1: Acyl-CoA oxidase 1
Acadl: Acyl-coenzyme A dehydrogenase, long-chain
CPT-1: Carnitine palmitoyltransferase-1
Ehhadh: Enoyl-CoA, hydratase/3-hydroxyacyl CoA dehydrogenase
Fgf21: Fibroblast growth factor 21
eWAT: Epididymal white adipose tissue
Cidea: Cell death–inducing DFFA-like effector a
Ucp1: Uncoupling protein 1
Hsl: Hormone-sensitive lipase
Atgl: Adipose tissue triglyceride lipase
CrebH: cAMP-responsive element-binding protein H
FFA: Free fatty acid
RXR: Retinoid X receptor
PPRES: PPAR response element
BAT: Brown adipose tissue
H&E: Hematoxylin and eosin
PBS: Phosphate-buffered saline
qRT-PCR: Quantitative real-time PCR.
